# Automated detection of near falls: algorithm development and preliminary results

**DOI:** 10.1186/1756-0500-3-62

**Published:** 2010-03-05

**Authors:** Aner Weiss, Ilan Shimkin, Nir Giladi, Jeffrey M Hausdorff

**Affiliations:** 1 Laboratory for Gait & Neurodynamics & Movement Disorders Unit, Tel-Aviv Sourasky Medical Center, Tel-Aviv, Israel; 2 Dept of Physical Therapy, Sackler Faculty of Medicine, Tel-Aviv University, Tel-Aviv, Israel; 3 Harvard Medical School, Boston, MA, USA; 4 Dept of Electrical Engineering, Tel Aviv University, Tel-Aviv, Israel; 5 Dept of Neurology, Sackler Faculty of Medicine, Tel-Aviv University, Tel-Aviv, Israel

## Abstract

**Background:**

Falls are a major source of morbidity and mortality among older adults. Unfortunately, self-report is, to a large degree, the gold-standard method for characterizing and quantifying fall frequency. A number of studies have demonstrated that near falls predict falls and that near falls may occur more frequently than falls. These studies suggest that near falls might be an appropriate fall risk measure. However, to date, such investigations have also relied on self-report. The purpose of the present study was to develop a method for automatic detection of near falls, potentially a sensitive, objectivemarker of fall risk and to demonstrate the ability to detect near falls using this approach.

**Findings:**

15 healthy subjects wore a tri-axial accelerometer on the pelvis as they walked on a treadmill under different conditions. Near falls were induced by placing obstacles on the treadmill and were defined using observational analysis. Acceleration-derived parameters were examined as potential indicators of near falls, alone and in various combinations. 21 near falls were observed and compared to 668 "non-near falls" segments, consisting of normal and abnormal (but not near falls) gait. The best single method was based on the maximum peak-to-peak vertical acceleration derivative, with detection rates better than 85% sensitivity and specificity.

**Conclusions:**

These findings suggest that tri-axial accelerometers may be used to successfully distinguish near falls from other gait patterns observed in the gait laboratory and may have the potential for improving the objective evaluation of fall risk, perhaps both in the lab and in at home-settings.

## Background

Falls are a significant cause of morbidity and mortality, especially among older adults and many patient populations [1]. In June of 2009, research on falls among the elderly was listed as the 3^rd ^item in the top priority group in the Institute of Medicine's report to Congress on national priorities for the United States [2]. Much effort has been devoted to the development of methods for evaluating fall risk [3-6], but the most common means of quantifying falls remains self-report. Despite its widespread use, this method has three key limitations: 1) it is subjective in nature, relying on subjects' motivation and memory (which can be problematic in its own right), 2) it requires a long observation period (e.g., typically six months or more), and 3) sensitivity may be lacking (e.g., subjects are typically classified as fallers and non-fallers, but the absence of a more refined scale may limit sensitivity and the ability to evaluate intervention efficacy).

Different techniques have been developed to automatically identify falls and related measures with varying degrees of success using sensors embedded in the home environment or body-fixed sensors [7-9]. Body-worn fall-detection systems are intended for long-term, automated detection of activity, in general, and falls, more specifically [5,10-16]. For example, methods based on accelerometry have been proposed as being suitable for the detection of falls in ambulatory subjects [5,7,14,17-19]. These methods have been developed as part of alarm and similar warning systems in order to automatically identify an older adult who has experienced a fall and is in need of immediate assistance. This approach is appropriate for alarm and related applications and has, indeed, been used successfully for these purposes and for the assessment of activity. It is, nonetheless, important to keep in mind that a community-living older adult typically falls less than two times per year [20] and that falls, at least among the healthy elderly, are relatively rare events, albeit with very significant consequences. Thus, continuous monitoring of falls using body-fixed sensors would generally require very long periods of observation (e.g., half a year) to capture a fall incident, minimizing the practicality and feasibility of using this approach to quantify fall risk by measuring actual falls.

We hypothesized that methods based on body-fixed sensors could, however, be adapted to identify missteps or near falls, potentially enhancing the utility of an approach for assessing fall risk. Missteps and near falls are used here synonymously as a stumble or loss of balance that would result in a fall if sufficient recovery mechanisms were not activated [21]. Automatic identification of near falls should, a priori, provide objective quantification of a sensitive marker of fall risk, perhaps over a shorter observation time periods. Indeed, a number of studies have found that near falls based on self-report are related to fall risk [21-27], that near falls are more frequent than falls [21-23,25,27,28], and that near falls may occur before falls [23-25], enhancing the potential predictive value of near falls. These properties indicate that near falls are clinically relevant markers of falls worthy of further study. Objective techniques for quantifying these events are, however, lacking [21]. The primary aim of the present study was to begin to develop and assess signal processing methods for detecting nearfalls using body-fixed sensors.

## Methods

### Subjects

Young adults (n = 10; ages: 22-28 yrs, 4 males) and older adults (n = 5; ages: 63-77 yrs, 3 males) participated in this study. Subjects in both groups were healthy and had no gait disturbances. Subjects were excluded if they had any disability likely to impair gait or balance, cognitive decline, or dementia (Mini Mental State Exam score<24). The research carried out on humans was in compliance with the Helsinki Declaration and the study protocol was approved by Human Studies Committee of the Tel Aviv Sourasky Medical Center. All subjects provided informed written consent.

### Procedures

**Figure 1:**
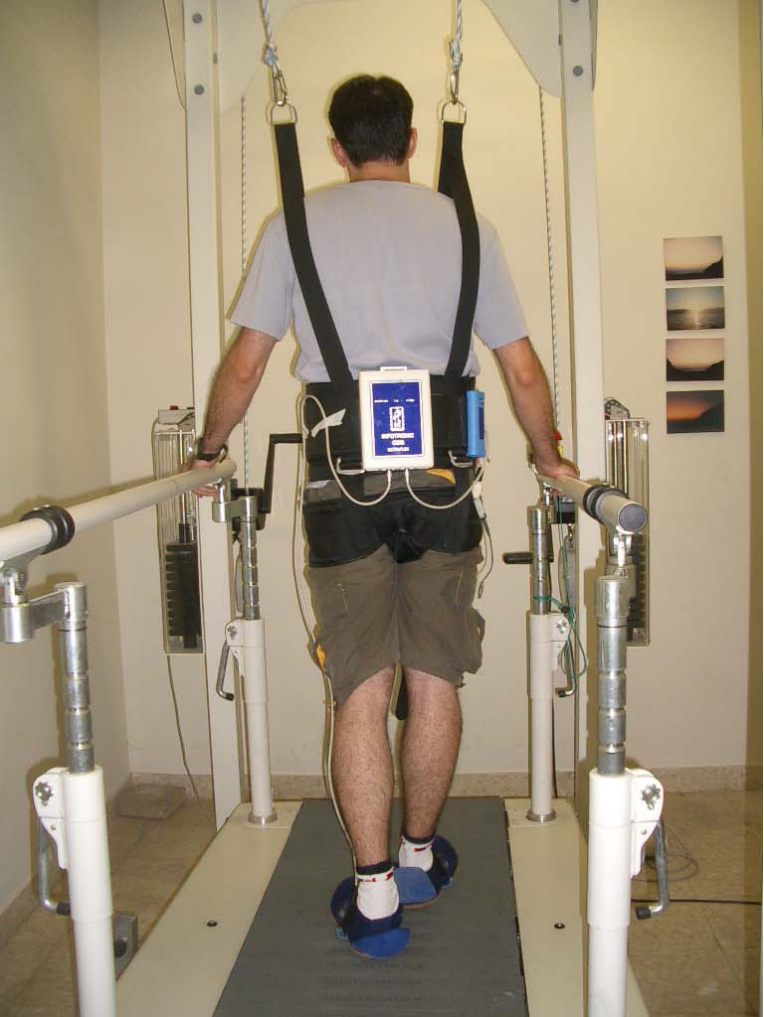
**The setup used to assess near falls**. The medical treadmill and harness used are shown along with the sensors used. A 3D accelerometer is located on the lower back and held in place by the large belt shown.

**Figure 2:**
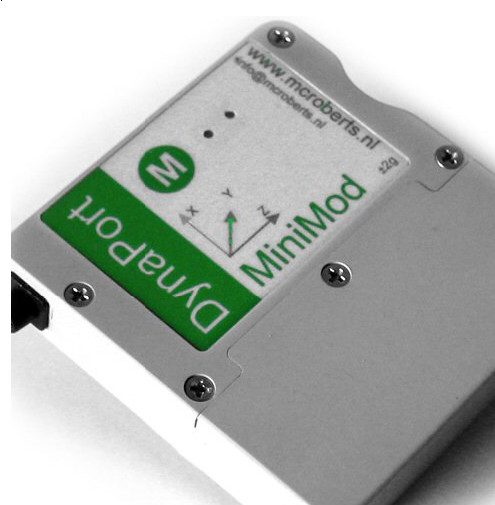
**The accelerometer used in the present study**. A DynaPort portable tri-axial accelerometer (McRoberts, The Hague, NL) was placed on the lower back to measure accelerations, the input to the near falls algorithms.

Subjects walked on a medical treadmill equipped with a safety harness to prevent actual falls (see Figure 1) at three different paces (i.e., self-selected slow, normal and fast). At each pace, subjects walked for 2 minutes without obstacles and 2 minutes with obstacles, randomly placed in the subject's path every few seconds, but not in the line of vision, to induce near falls. Obstacles included empty shoeboxes (30 cm × 20 cm × 12 cm), shoeboxes filled with stones, empty carbon cylinder rolls (90 cm height and 8 cm diameter), and ropes. A sheet was placed just in front of the subject, between the subject's face and the floor, to hide the presence of the obstacles from the subject's view of the floor and the obstacles placed on the treadmill. Thus, the subjects could not see the obstacles. Observational analyses were used to define near falls (a loss of balance that would have resulted in a fall if corrective measures were not taken). Other gait irregularities included stepping over or kicking obstacles were not defined as a near fall. The decision to annotate a given segment as a near fall (or not) was made in real-time by an observer, without knowledge of the accelerometer data (i.e., blinded to this data). A DynaPort^® ^MiniMod portable tri-axial accelerometer (McRoberts, The Hague, NL) was worn on the lower back to measure the vertical, anterior-posterior and medio-lateral accelerations (see Figure 2). The accelerometer range was ± 2 g and its sampling frequency was 100 Hz.

### Data Analysis

The data was processed using Matlab (the MathWorks Inc). All gait intervals were divided into 5 second, non-overlapping segments. The normal gait segments were compared to the "near fall" segments. Acceleration-axis calibration was performed, as described previously [10], in order to correct for possible axis-tilt due to the orientation of the device on the subject or due to lower back tilt of the subject. The acceleration axes were calibratedto match the orthogonal axes. Afterwards, the anterior-posterior acceleration was low passed filtered (a 1 Hz cutoff frequency FIR filter was used). Step cycles were defined as the zero crossings of the filtered signal [29] and the intervals between each two successive steps determined the step cycle time series.

**Figure 3:**
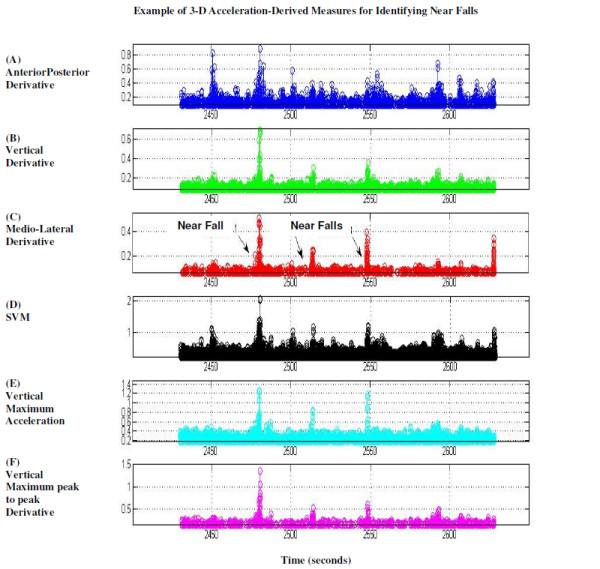
**Acceleration derived signals during 3 near falls, as labeled, and during other gait intervals**. A-C) Derivatives in three axes. D) Signal Vector Magnitude, calculated as the root of the square sums of the 3 axes acceleration signals, E) Vertical Maximal acceleration. F) Vertical maximum peak-to-peak acceleration derivative. All signals display indications of the near falls.

For each 5 second gait segment, the signal vector magnitude (SVM),

and the Normalized Signal Magnitude Area (SMA),

where x,y,z are the 3 axes of acceleration, were determined [11] and thresholds were used to define a near fall. The SVM provides a measure of the degree of movement intensity [11]. Other derived parameters included the acceleration derivative (jolt), maximum acceleration amplitude (Max), the maximum acceleration derivative (Maxdiff), the maximum peak-to-peak acceleration amplitude (Maxp2p) and the maximum peak-to-peak acceleration derivative (Maxp2pdiff), defined as the difference between the maximum and minimum acceleration derivatives (see, for example, Figure 3). The standard deviation of the signal in each gait interval was also determined. In addition, step regularity, stride regularity, and symmetry were calculated, as previously described [30].

#### Algorithm Assessment

For each gait parameter, the best threshold for distinguishing between normal gait segments (i.e., epochs) and near falls was determined by plotting a range of possible thresholds using a Receiver Operating Characteristic curve, ROC, and choosing the threshold with the best specificity and sensitivity values, i.e., the ones closest to the (0,1) point. The algorithm performance was examined for each parameter separately and for two or three parameter combinations. For the multiple parameter combinations, we checked detection performance when all parameters were above a certain threshold, i.e., the "and" state, and when at least one parameter was above a certain threshold, i.e., the "or" state. Performance was expressed by means of sensitivity and specificity.

## Results

Overall, we observed 592 normal segments, 21 near falls, 18 stops, 30 step-overs (stepping over obstacles), and 28 kicks. All of the acceleration derived measures showed higher values during a near fall (e.g., see Figure 3). The best single parameter indicator for a near fall was the vertical maxp2pdiff: it achieved a sensitivity of 85.7% and specificity of 88.0% for identifying near falls. Other measures were also fairly successful at identifying near falls (see Table 1). The best 2-parameter indicator for a near fall was the "and" combination of the vertical maxp2pdiff along with the vertical maximum: sensitivity of 85.7% and specificity of 90.1% (see Table 2). Results were slightly better when extracting all the irregular intervals (e.g., kicks/stops/step-overs) from the "normal" group (data not shown).

**Table 1:** Sensitivity and specificity for detecting near falls using single parameters.

Parameter	axis	NOT Near Fall mean	Near Fall mean	NOT Near Fall SD	Near Fall SD	threshold	Sensitivity (%)	Specificity (%)	Detection (%)**
Max*	**V**	**0.45**	**0.83**	**0.20**	**0.30**	**0.56**	**90.48**	**81.89**	**20.46**

Max	M-L	0.31	0.60	0.16	0.26	0.41	71.43	86.38	31.65

Max	A-P	0.33	0.48	0.17	0.22	0.38	52.38	73.80	54.35

Maxdiff	V	0.17	0.37	0.09	0.15	0.21	80.95	80.99	26.91

Maxdiff	M-L	0.15	0.36	0.10	0.26	0.17	76.19	75.60	34.09

Maxdiff	A-P	0.19	0.41	0.19	0.24	0.24	76.19	82.34	29.64

maxp2p	V	0.71	1.40	0.29	0.53	0.88	90.48	80.39	21.80

maxp2p	M-L	0.63	1.35	0.30	0.67	0.80	80.95	83.53	25.18

maxp2p	A-P	0.75	1.38	0.43	0.57	0.93	76.19	82.93	29.30

**maxp2pdiff**	**V**	**0.34**	**0.69**	**0.19**	**0.26**	**0.48**	**85.71**	**88.02**	**18.65**

maxp2pdiff	M-L	0.30	0.72	0.20	0.52	0.35	80.95	76.95	29.90

maxp2pdiff	A-P	0.42	0.86	0.36	0.48	0.52	80.95	81.59	26.49

Std	V	0.13	0.19	0.06	0.05	0.16	61.90	74.55	45.82

Std	M-L	0.11	0.16	0.04	0.05	0.13	66.67	79.94	38.90

Std	A-P	0.13	0.17	0.05	0.04	0.15	61.90	79.34	43.34

Step regularity	V	0.58	0.32	0.17	0.12	0.27	71.43	5.99	98.26

Stride regularity	V	0.54	0.27	0.16	0.15	0.22	61.90	5.69	101.72

Symmetry	V	1.13	1.36	0.39	0.57	1.24	61.90	79.79	43.13

The sensitivity and specificity values obtained for detecting near falls (n = 21) as compared to non-near falls (n = 668) using single parameters. Non-near falls included regular gait intervals combined with the irregular (non-near falls) intervals (e.g., kicks, stepovers, stops).

*Max = maximum acceleration amplitude; Maxdiff = maximum acceleration derivative; Maxp2p = maximum peak-to-peak acceleration amplitude; Maxp2pdiff = maximum peak-to-peak acceleration derivative; Std = standard deviation; V: vertical; M-L: medio-lateral; A-P: anterior-posterior.

** The detection % is the distance from the ideal ROC curve (the lower, the better).

**Table 2:** Sensitivity and specificity for detecting near falls using multiple parameters.

Parameters*	State	Sensitivity (%)	Specificity (%)	Detection (%)**
Max-V, Maxp2pdiff-V	and	85.71	90.12	17.37

[Max-V, Maxp2p-V, Maxp2pdiff-V	and	85.71	90.12	17.37

Maxp2p-V, Maxp2pdiff-V	and	85.71	89.37	17.81

[Max-V, Maxp2p-V	and	90.48	84.13	18.51

Max-M-L, Maxp2pdiff-V	or	90.48	81.74	20.59

Maxp2p-M-L, Maxp2pdiff-V	or	95.24	79.49	21.06

Max-V, Maxdiff-V, Maxp2pdiff-V	and	80.95	90.42	21.32

Maxdiff-V, Maxp2p-V, Maxp2pdiff-V	and	80.95	89.82	21.60

Maxp2pdiff-V, Maxp2pDiff-A-P	or	95.24	78.74	21.79

Maxdiff-A-P, Maxp2pdiff-V	or	95.24	78.59	21.93

The sensitivity and specificity values obtained for detecting near falls (n = 21) as compared to non-near falls (n = 668) using multi-parameter combinations Non-near falls included regular gait intervals combined with the irregular, non-near falls intervals (e.g., kicks, stepovers, stops).

*The parameter combinations for the algorithm's detection criterion included single parameters, and multiple parameters. For the multiple parameter combinations, we checked the case of passing the detection criterion for all parameters (state **"and"**), versus passing the detection criterion for at least one parameter (state **"or"**).

Max = maximum acceleration amplitude; Maxdiff = maximum acceleration derivative; Maxp2p = maximum peak-to-peak acceleration amplitude; Maxp2pdiff = maximum peak-to-peak acceleration derivative; Std = standard deviation; V: vertical; M-L: medio-lateral; A-P: anterior-posterior.

**The detection% is the distance from the ideal roc curve (the lower, the better). For brevity, the results are shown for only the best 10 combinations.

## Discussion

The results of the present study demonstrate that a single accelerometer may be placed on the trunk of an individual to automatically distinguish near falls from other stepping patterns, with reasonable sensitivity and specificity. Interim analysis of a follow-up study among elderly fallers and non-fallers who walked over-ground also support the idea that these objectively identified near falls are more common among older adults with a history of falling, consistent with the results of self-report studies of near falls [21-28]. Taken together, these findings suggest that perhaps long-term recordings and measurement of near falls, as subjects carry out activities of daily living, is likely to be a clinically relevant, objective adjunct measure of fall risk, possibly improving sensitivity and reducing the observation time required.

This preliminary study has several limitations. Ongoing studies are designed to examine how the developed algorithms work in real-world conditions and to evaluate the predictive value of acceleration-derived measures of near falls in different control and patient populations (e.g., patients with neurodegenerative disease) and in aging (e.g., young vs. older adults). Normal balance responses may have been altered and restricted by the treadmill setup (e.g., the treadmill provides bars for support and promotes continuous walking). In this initial study, we focused on the identification of near falls, but made no attempt at differentiating between the loss of balance and the recovery. Theoretically, these are two distinct processes. In this work, we aimed to identify the "stumble" or near fall. It is, however, possible that some of the recovery phase may have been identified, although most of the derived parameters that had good success in identifying these events are, at least intuitively, more likely related to the loss of balance than to the recovery process. Further work is also needed to verify that the developed algorithms are successful at identifying near falls in normal and free walking environments. There are some subtle differences between over-ground walking and treadmill walking and, as a result, the accelerometer signal is not identical in both conditions. However, even on a treadmill, the signals from all three axes have a form that is similar to over-ground walking, supporting the idea that over-ground near falls can also be detected using the algorithms described.

Despite these and other limitations, the initial results reported here motivate continued work along these lines, provide a basis for future studies in both in the lab and at-home settings, and suggest that a tri-axial accelerometer can successfully identify near falls and may also have the potential for improving the objective evaluation of fall risk.

## Competing interests

A patent related to this work has been submitted.

## Authors' contributions

AW and IS designed the study and carried out the data collection and analysis. AW drafted the manuscript. NG assisted with the study design and manuscript revision. JMH assisted with study design, data analysis and manuscript revision. All authors read and approved the final manuscript.
